# Spontaneous Resolution of Retinal Pigment Epithelial Detachments and Visual Improvement in Patient with MPGN II: A Case Report

**DOI:** 10.1155/2012/864198

**Published:** 2012-12-05

**Authors:** T. Empeslidis, U. Imrani, A. Vardarinos, N. Menassa, S. Banerjee

**Affiliations:** Ophthalmology Department, Leicester Royal Infirmary, University Hospitals of Leicester, Leicester LE15WW, UK

## Abstract

A 31-year-old female suffering from membranoproliferative glomerulonephritis type II (MPGN II) presented to the Eye Casualty Department reporting a history of blurred and distorted vision. The patient appeared to have drusenoid retinal epithelial detachments and minimal intraretinal fluid. The subretinal deposits, basal lamina drusen, and pigment epithelial detachment appeared to resemble a “stars in the sky” picture with no symmetry between the eyes. The retinal pigment epithelial detachments improved and flattened over 18 month. and the best corrected visual acuity improved in the most affected eye. There was no evidence of neovascularization, and the intraretinal fluid disappeared spontaneously.

## 1. Introduction

We present a case of a 31-year-old female who attended our Eye Casualty Department reporting a 2-month history of blurred and distorted vision. This resulted in problems with near vision tasks such as reading and working on the laptop. The patient had her medical and ocular history taken, revealing a long standing history of membranoproliferative glomerulonephritis type II (MPGN II) which had been confirmed with renal biopsy. The condition manifested at 10 years of age with nephritic type symptoms. The patient required a kidney transplant 6 years ago and was on a maintenance dose of 4 mg Prednisolone at the time she presented. There was no other relevant medical or ocular history. A full clinical examination of the eyes was performed as well as additional investigations. Blood tests including full blood count, urea, and electrolytes and liver function tests were performed in addition to ocular coherence tomography (OCT) and fluorescein angiography (FFA). 

## 2. Case Report

**Figure 1:**
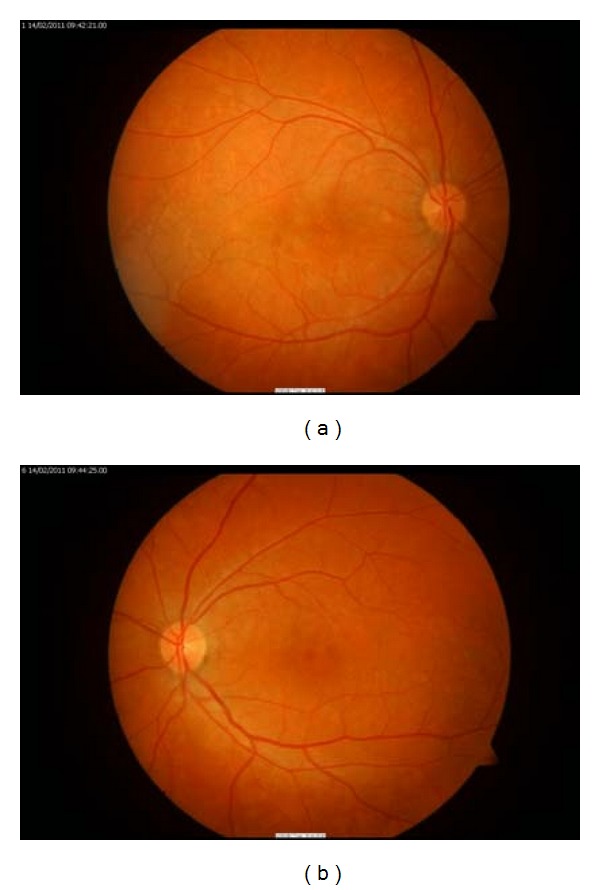
Fundus photographs at presentation.

The patient was assessed; the best corrected visual acuity of the right eye was 6/36 and left eye was 6/9. Funduscopic examination revealed clinical signs consistent with retinal pigment epithelial detachments and small drusen-like lesions with reasonably identified borders in the posterior pole. The right eye had far more drusen-like lesions compared to the left eye (Figure 1).

**Figure 2:**
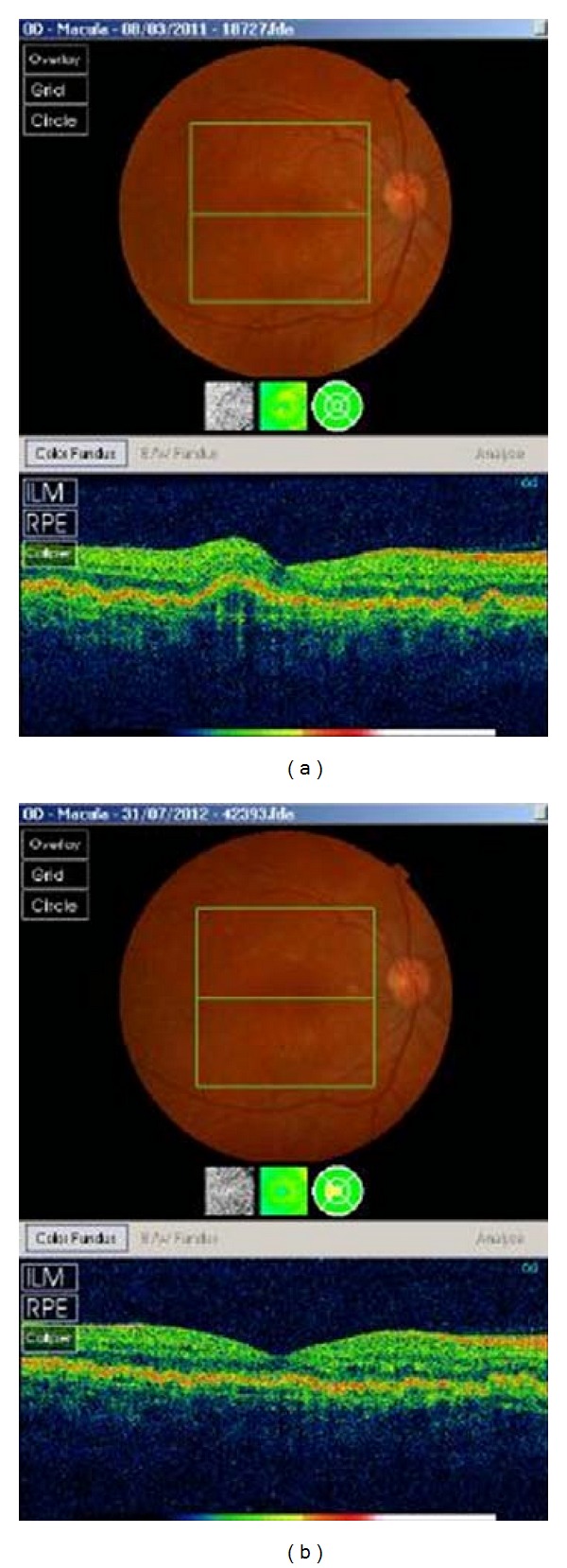
OCT images of the right eye at presentation (a) and 18 months later (b).

The patient had OCT examinations with the SOCT Copernicus (OPTOPOL Technology S.A., Poland) and Topcon 3D OCT-1000 (Topcon Medical Systems, Oakland, NJ) performed (Figure 2). On OCT there was evidence of spread retinal pigment epithelial detachments. The drusenoid retinal pigment epithelial detachments were more extensive, numerous, and taller in the right eye. There was also evidence of intraretinal fluid in a cystoid form of less than 50 *μ*m in the inner retina layers. And there were numerous small shallow retinal pigment epithelial detachments. There was no evidence of subretinal fluid in either eye, optically clear material underneath the drusenoid pigment epithelial detachment, or presence of back reflection from the inner segment/outer segment photoreceptor junction. The retinal pigment epithelium (RPE) was intact and there was no evidence of rupture. However, the RPE was more irregular in the right eye. The pigment epithelial detachment elevations were higher in the right eye where vision was worse.

**Figure 3:**
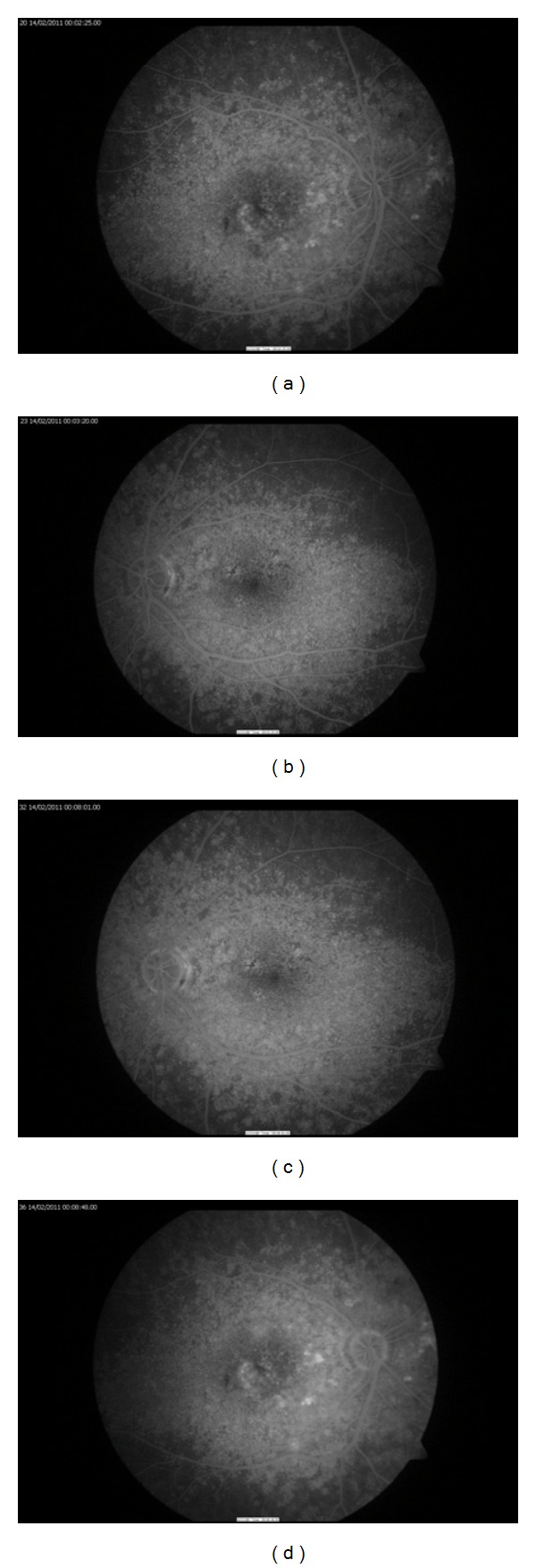
Fluorescein angiogram at presentation.

On FFA from early phases there was a window defect due to the basal lamina drusen. In late phases there was staining of hyperfluorescence; however, there was no evidence of leakage. FFA also showed that the retinal pigment epithelial layer was intact (Figure 3). 

We monitored the patient over 18 months and have observed flattening of the pigment epithelial detachments and an improvement in visual acuity from 6/36 to 6/9 in the right eye. The left eye maintained a visual acuity of 6/9 during the 18 months. During this period there was no reported worsening of the kidney condition and the patient remained on the low dose of Prednisolone. 

## 3. Discussion

MPGN II or dense deposit disease is a rare condition that can potentially affect the eyes and the ocular manifestations were first described by Duvall-Young et al. in 1989 [1]. It is the causative factor of deposition of electron dense material within the kidney's glomerular membrane and within the Bruch's membrane and the RPE choriocapillaris area [1, 2].

D'souza et al. documented the presence of deposits similar to basal lamina drusen in patients with MPGN II and it appears that the deposition of the drusen-like changes takes place early in the second decade of a patient's life [2]. No correlation between the extent of retinopathy and the severity of the renal disease exists [2]. However, some authors suggest a correlation between the development of eye changes and the duration of the renal disease [3].

Choroidal neovascularisation (CNV) and idiopathic central serous chorioretinopathy are complications recognised to be associated with MPGN II [4–6]. It has been suggested that factors other than drusen-like lesions alone lead to CNV development in MPGN II. These include the increased lipid content in the Bruch's membrane or the decreased choriocapillary density [2]. 

Over the 18-month period we followed the patient up; there was a significant improvement in the visual acuity. The clinical picture during this time showed flattening of the pigment epithelial detachments and increased RPE regularity which correlates with the improvement in acuity. There was also a decrease in intraretinal fluid; however, this does not fully explain the improvement in visual acuity. 

Our findings are the first to our knowledge to report significant improvement in the anatomical and clinical picture in the retina of patients suffering from MPGN II. Although a level of fluctuation is potentially expected, this case is remarkable due to the significant improvement in visual acuity from 6/36 to 6/9. We believe it is important to know that patients with MPGN-II-related eye disease may have considerable improvement in the clinical and anatomical picture. There was no comorbidity, change in the patient's medication, or other factors that may have led to the documented changes.

Patients with MPGN II require baseline assessment of their eyes at diagnosis as the formation of reticular drusen and choroidal neovascularisation is well established in the literature [7]. Regular assessment by a retinal specialist is also recommended for these patients. Treatment options of choroidal neovascularisation due to MPGN II by, for example, antivascular endothelial growth factors require evaluation to determine efficacy.
